# Information Drives Sensory Perception and Willingness-to-Pay for Partially Dealcoholized Wine: Evidence from a Between-Subjects Experiment in Italy

**DOI:** 10.3390/foods15122056

**Published:** 2026-06-06

**Authors:** Francesco Di Cosola, Alessandro Petrontino, Emanuela Tria, Stefano Mattia, Valentina Fanelli, Cinzia Montemurro, Francesco Bozzo

**Affiliations:** 1 Department of Soil, Plant and Food Science (Di.S.S.P.A.), University of Bari Aldo Moro, Via G. Amendola, 165/a, 70126 Bari, Italy; francesco.dicosola@uniba.it (F.D.C.); emanuela.tria@uniba.it (E.T.); stefano.mattia@uniba.it (S.M.); valentina.fanelli@uniba.it (V.F.); cinzia.montemurro@uniba.it (C.M.); francesco.bozzo@uniba.it (F.B.); 2 Sinagri s.r.l., Spin Off of the University of Bari “Aldo Moro”, 70126 Bari, Italy

**Keywords:** dealcoholized wine, sensory evaluation, consumer perception, knowledge, willingness-to-pay

## Abstract

The growing diffusion of dealcoholized wines calls for a deeper understanding of how information and sensory evaluation jointly shape consumer acceptance, particularly in traditional wine markets. This study investigates the effects of different combinations of information and tasting on sensory evaluation and willingness-to-pay for a partially dealcoholized red wine. A between-subjects experiment was conducted during a scientific festival in Apulia (Italy), where participants were assigned to three conditions (INFO-SENS, SENS, INFO) and evaluated a non-commercialized Apulian Primitivo (1.5% ABV), produced through low-temperature vacuum evaporation and not pasteurized. Sensory attributes (visual, olfactory, gustatory, overall) were rated on 9-point hedonic scales, and WTP for a 125 mL glass was elicited using a payment card. The results show that the information-only group (INFO) reported the highest WTP, compared to the tasting-only group (SENS). Information exposure increased visual and olfactory evaluations, but not gustatory ratings. Prior knowledge of NoLo wines was associated with negative expectations, though this effect was attenuated by information. Overall, within this experimental setting, information emerged as a key driver of perceived value outweighing sensory liking, although the two remained positively correlated. These findings highlight the importance of transparent communication in fostering acceptance and repositioning dealcoholized wine as a credible category within traditional markets.

## 1. Introduction

### 1.1. NoLo Wines: Market Dynamics, Technology, and Consumer Acceptance

The beverage sector is shifting toward healthier and sustainability-focused consumption patterns [1], impacting the wine industry where traditional Mediterranean markets face declining domestic demand [2,3]. Meanwhile, non-traditional markets in northern Europe, North America, and Asia are experiencing expanding consumption, driven by wellness and mindful drinking lenses [4,5]. The No–Low alcohol segment ranks among the fastest-growing beverage categories globally, with notable growth in the United Kingdom and United States [6,7]. In Europe, northern countries (e.g., the Netherlands, Finland, Germany), show faster increases in penetration, while established markets like France, Italy, and Spain still display higher absolute volumes but lower NoLo adoption rates [8,9]. Cross-country differences are amplified by consumption frequency patterns, which create greater heterogeneity in consumer preferences in traditional markets relative to northern European ones [10].

At the core of this dynamic lies fully or partially dealcoholized wine, defined as a fermented grape product from which ethanol has been removed to achieve an alcohol by volume (ABV) below 0.5% for dealcoholized wine, or above 0.5% vol and below the original category minimum alcoholic strength for partially dealcoholized wine, in line with EU Regulation 1308/2013, supplemented and amended by Regulation (EU) 2021/2117 [11].

Unlike non-fermented alternatives such as juices or soft drinks, NoLo wine can retain aromatic complexity and therapeutic bioactive profiles, including phenolic compounds like resveratrol and quercetin [12,13]. Early dealcoholization methods often compromised sensory integrity [14], but modern techniques like vacuum distillation (VD), reverse osmosis (RO), and spinning cone columns (SCC) have minimized organoleptic losses [8,15]. For instance, Belisario-Sánchez et al. [16] reported that SCC treatment preserved, and in some cases slightly increased, the levels of resveratrol and flavonoids, likely due to a concentration effect. Similarly, Schmitt et al. [17] found that modern dealcoholization processes can preserve key aroma characteristics in white wine, including fruity notes such as apple, citrus, and peach. Despite these advances, consumers still perceive alcohol as a key contributor to wine quality, body, and authenticity [18].

Pioneering studies on NoLo wines laid the foundation for understanding the relationship between alcohol content, sensory characteristics, and consumer acceptance [19,20] while later research highlighted how psychological, social, and moral perceptions surrounding these products have evolved over time [21]. Motivations to try NoLo wines include epistemic and emotional drivers such as curiosity, novelty, or the search for alternatives that allow wine enjoyment without alcohol [22]. Health motivations emerge as a key aspect in some markets, with Fuentes-Fernández and del Campo-Villares [23] reported that over 60% of Spanish consumers cite it as the primary motivator for trying dealcoholized wine, especially among millennials and women showing high openness to adoption. In Australia, however, Saliba et al. [21] found no direct link between health orientation and interest in low-alcohol wine, while Bucher et al. [24] observed positive blind-tasting evaluations but a reluctance to pay standard prices. Nevertheless, skepticism remains particularly strong in traditional markets such as Italy, where the central role of wine in national identity reinforces cultural and economic resistance to dealcoholized wine, leading consumers to demand price discounts proportional to reductions in alcohol content [25,26].

### 1.2. Information Cues, Sensory Perception, and Italian Market Resistance

Academic research consistently demonstrates that acceptance of innovative food and wine products depends on a complex interplay between extrinsic cues (e.g., labels and production information) and intrinsic sensory perception. Expectations shaped by non-sensory information, such as origin or production details, can reshape hedonic ratings and consumption behavior [27,28,29]. For dealcoholized wines, low-alcohol labeling can alter sensory appreciation even in blind tastings [30], and for unfamiliar products cumulative information provision tends to increase expectations, liking, and willingness-to-pay (WTP), with effects that vary across consumer demographics [31,32]. Written information and clear labeling enhance acceptance, particularly among informed consumer segments [33] and contribute to shaping the perceived acceptability of low-alcohol wines [34]. Alcohol content remains a key determinant of perceived quality and price in beverages, while ambiguous or overly technical labels (for example “alcohol-free”) may evoke perceptions of artificiality and erode trust [35]. The reduction in alcohol content can also trigger a symbolic devaluation of the product even when sensory appreciation remains acceptable [24]. As a result, consumer acceptance remains highly context dependent [36]. This challenge is further intensified by regulatory variations across jurisdictions, which complicate consumer interpretation and reduce overall confidence in the product category [37]. An emblematic case emerges even in the non-Western Muslim market, where the term “dealcoholized wine” can be perceived as an unhealthy or forbidden product, illustrating how cultural context shapes interpretation [38].

Italy provides an ideal context for studying NoLo acceptance, as cultural norms continue to privilege traditional wine in terms of production, consumption and identity, while national regulatory authorities (DM 6728/16, 20 December 2024) have only recently enabled domestic production under stricter conditions than many European peers [39]. Within this setting, nutrition-oriented consumers appear more responsive to detailed labeling and health-related claims, whereas more socially oriented drinkers are less likely to consult labels and show weaker preferences for low-alcohol alternatives [40,41]. This combination of entrenched tradition, regulatory delays, and low category familiarity makes Italy an important case of a conservative market, providing a compelling setting for examining consumer evaluations [42]. Although the literature on Italian dealcoholized wine preferences has expanded substantially using diverse methods, the interplay of strategic product information, sensory assessment, and price remains underexplored. Sensory analysis combined with consumer research offers essential tools for new product development, optimizing quality and market adoption for innovative beverages [43].

### 1.3. Research Objectives and Hypotheses

Motivated by regulatory innovations, technological advances, and shifting consumer attitudes, this study examines how sensory ratings and product information interact and jointly affect economic behavior toward dealcoholized red wine among a consumer sample of Italian consumers. Specifically, we address the following research questions:

• RQ1: How does product information provided during tasting evaluation experiments influence sensory perceptions and WTP for partially dealcoholized wine?
• RQ2: How are sensory perceptions and WTP linked to prior NoLo knowledge, wine habits and socio-demographic characteristics?

To address these questions, we compare three experimental consumer group evaluations varying in information exposure and tasting sequence. In particular, the research investigates whether transparent communication regarding production methods and product composition can enhance acceptance in a culturally conservative market.

Based on this framework, three testable hypotheses (H1–H3) are proposed:

• H1: Product information positively influences sensory evaluations and willingness-to-pay for dealcoholized wine.
• H2: Information moderates the relationship between prior NoLo wine knowledge on sensory evaluation and willingness-to-pay.
• H3: Consumer habits and socio-demographic characteristics influence sensory evaluation and willingness-to-pay for dealcoholized wine.

## 2. Materials and Methods

### 2.1. Experimental Design and Participants

This study employed a between-subjects experimental design to investigate how product information and tasting experience jointly influence consumers’ acceptance of partially dealcoholized red wine. The experiment was conducted during a scientific festival in Puglia, Italy (September 2025), where trained research assistants recruited participants and directed them to three separate evaluation stations. Participation was voluntary for adults aged 18 years or older with sufficient Italian-language proficiency. Individuals were excluded if they reported allergies, pregnancy, or conditions affecting taste or smell perception. Professional sommeliers were also excluded to reduce expert-related bias in sensory evaluation. After providing written informed consent for the tasting of an uncommercialized food product, the participants were randomly assigned to one of three groups, with allocation managed to maintain broad balance by age and gender across conditions. Given the event-based recruitment setting, the sample represents a convenience sample rather than a representative sample of Italian wine consumers. However, this real-world public environment was suitable for exploring consumer reactions to an innovative wine product among individuals potentially interested in food innovation.

### 2.2. Experimental Procedure

**Figure 1:**
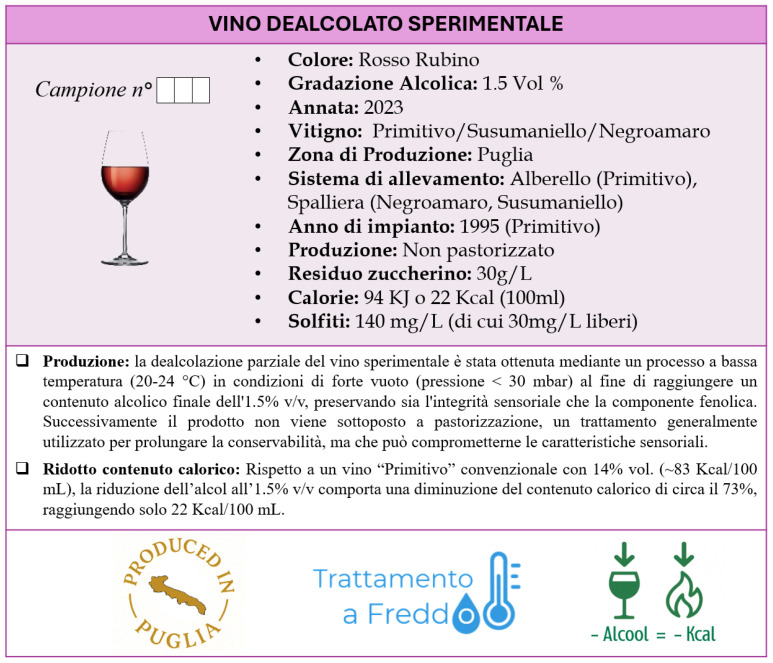
The standardized information sheet detailing the product’s attributes. Note: English version—“Experimental Dealcoholized wine: Sample no.; Color: Ruby Red; Alcohol content: 1.5% Vol; Vintage: 2023; Grapes: Primitivo/Susumaniello/Negroamaro; Production area: Apulia; Training system: bush-trained (Primitivo) and trellised; Planting year: 1995 (Primitivo); Production: Not pasteurized; Residual sugar: 30 g/L; Calories: 94 kJ or 22 Kcal (100 mL); Sulphites: 140 mg/L (of which 30 mg/L free); Production: The partial dealcoholization of the experimental wines was performed using low temperature (20–24 °C) under high vacuum conditions (pressure < 30 mbar) technology to achieve a final ethanol content of 1.5% *v*/*v* while preserving sensory and phenolic integrity. Subsequently the product is not subjected to pasteurization, a treatment generally used to extend shelf life, but which can reduce sensory characteristics.; Reduced caloric content: Compared with a conventional ‘Primitivo’ wine with 14% ABV (~83 Kcal/100 mL), reducing alcohol to 1.5% *v*/*v* yields only 22 Kcal/100 mL a ~73% decrease in caloric content”.

All the participants first completed a short questionnaire covering socio-demographic characteristics, wine consumption habits, color preferences, prior NoLo wine awareness, and previous tasting experience. They were then exposed to one of three experimental conditions, differing in the timing of product information, tasting and economic valuation. The participants in the INFO-SENS group received standardized product information before tasting the wine and then completed the sensory evaluation and WTP task. The participants in the SENS group tasted the wine without prior product information and then completed the sensory evaluation and WTP task. The participants in the INFO group received the same standardized information sheet describing the wine’s main characteristics (Figure 1) and stated their WTP based on information alone before tasting the wine, which was offered and evaluated only afterward. The information sheet included production details typically required on wine labels (ABV, origin, grape varieties, sulfites) supplemented by NoLo-specific technical information and health-related information that previous research has shown to be particularly relevant for NoLo acceptance. In contrast, the SENS group received only the minimum instructions necessary to complete the tasting task.

The wine sample was an uncommercialized and anonymized partially dealcoholized red wine from 100% Apulian grapes (Primitivo, Susumaniello, and Negroamaro). Following fermentation, ethanol was removed using low-temperature vacuum evaporation (20–24 °C) under high-vacuum conditions (<30 mbar), in order to preserve aromatic integrity and limit organoleptic losses. According to the product specifications, the wine had 1.5% ABV, 30 g/L residual sugar, 140 mg/L sulfites, and an energy content of 22 kcal/100 mL. Following dealcoholization, microbiological stability was ensured through microfiltration, avoiding the pasteurization process in order to preserve the sensory characteristics of the product.

Consumer sensory evaluation was conducted outdoors in natural light using transparent 50 mL plastic glasses. The participants rated four sensory dimensions—visual, olfactory, gustatory, and overall assessment—on 9-point hedonic scales adapted from Italian Wine Tasters Organization (ONAV) descriptors and suitable for large consumer panels. For each dimension, the respondents provided a single holistic score ranging from 1 (“extremely dislike”) to 9 (“extremely like”), which incorporates the constituent attributes specified by ONAV—Clarity, Tone and Intensity for the visual dimension; Cleanliness, Intensity and Harmony for the olfactory dimension; and Body, Balance and Persistence for the gustatory dimension—rather than separate analytical assessments for each descriptor. Accordingly, the sensory evaluation should be interpreted as a consumer test conducted under naturalistic rather than laboratory-controlled conditions. After tasting, receiving the information or both depending on the experimental conditions, the participants indicated their purchase intention recorded into a binary indicator (yes/no). Positive responses prompted WTP elicitation for a 125 mL glass using a payment card format on ordinal scale range from EUR 4 to EUR 10, whereas the participants with a negative purchase intention were assigned a WTP value of EUR 0 generating a left-censored distribution structure.

### 2.3. Statistical Analysis

Descriptive statistics summarized socio-demographic and wine-related sample characteristics. All statistical analyses were performed using JASP version 0.19.3, an open-source software for statistical analysis. To assess baseline comparability across the experimental groups (INFO-SENS, SENS, INFO), chi-square (χ^2^) tests were conducted on categorical variables (gender, age group, citizenship, wine consumption, wine preferences, prior NoLo knowledge and tasting experience). Subsequently, depending on variable distribution and scale properties, one-way ANOVA and the Kruskal–Wallis test were employed to examine differences across experimental conditions, followed by appropriate post hoc comparisons. Tukey’s HSD test was performed when the assumption of homogeneity of variances was satisfied, whereas the Games–Howell procedure was applied in cases of unequal variances [44].

ANOVA assumes normality of residuals and homogeneity of variances, which were assessed using Levene’s test. When this assumption was violated, Welch-corrected F-tests were adopted as a robust alternative. In the presence of markedly non-normal distributions, non-parametric methods such as the Kruskal–Wallis test are typically preferred. However, previous evidence suggests that, in group comparisons, Welch’s ANOVA may remain a robust option even when residuals deviate from normality, including in cases where variances are homogeneous [45]. For these reasons, one-way ANOVA models with Welch correction were used to test mean differences across experimental conditions for sensory ratings, while Kruskal–Wallis and Dunn’s post hoc pairwise comparisons were used for the WTP variable.

A composite sensory liking score (hereafter Liking) was computed as the mean of visual, olfactory, gustatory, and overall assessment after verifying the internal consistency reliability of this four-item scale was acceptable, as indicated by Cronbach’s alpha (α = 0.85) exceeding the commonly recommended threshold of 0.70 [46]. To examine the effects of information provision (INF), prior NoLo knowledge (KNO), and their interaction (INF × KNO) on sensory acceptance, a two-way ANOVA was conducted on Liking. To examine the association between Liking and consumer characteristics, a linear regression model was estimated with Liking as the dependent variable and socio-demographic characteristics and wine-related variables as predictors. To assess the socio-demographic and behavioral determinants of WTP, a Tobit regression model was employed. This approach is appropriate when the dependent variable is left-censored at zero, a structural feature of WTP elicited via payment card adopted from [47,48]. For this reason, the model was restricted to the participants (n = 155) who expressed positive purchase intention (WTP > 0).

The results presented in the following sections are organized by outcome, respectively sensory and economic valuation. They follow the methodological approach described above in three steps closely aligned with the research questions and hypotheses. First (H1), we report between-group comparisons across experimental conditions. Second (H2), we examine the interaction between information provision and prior NoLo knowledge on Liking and WTP. Third (H3) we explore the contribution of socio-demographic and behavioral factors on Liking and WTP.

## 3. Results

### 3.1. Sample Characteristics

The final sample consisted of 187 participants across three experimental groups: INFO-SENS (n = 59), SENS (n = 60), and INFO (n = 68). The participants were predominantly young adults (63.1% aged 18–44 years), Italian nationals (87.7%), regular wine consumers (81.3%), and reported preference for red wine (53.5%). Most had no prior NoLo wine knowledge (72.2%) and no prior tasting experience (89.3%) (Table 1). Chi-square tests showed no significant difference between the groups for gender, age range, wine color preference, prior NoLo knowledge, or prior NoLo tasting, indicating satisfactory baseline comparability for subsequent outcome analyses of sensory evaluation and willingness-to-pay across the three experimental conditions. Significant differences emerged only for wine consumption status and non-EU citizenship.

### 3.2. Sensory Evaluation Across Experimental Conditions

One-way ANOVA conducted on sensory ratings revealed significant group differences for visual, olfactory and overall rating, but not for gustatory (*p* > 0.10). Post hoc comparisons showed that the informational treatment was associated with higher sensory evaluations in selected dimensions of dealcoholized wine. More specifically, across all sensory attributes, the INFO-SENS group reported the highest mean values, followed by the INFO group, whereas the SENS group exhibited the lowest scores along with the largest standard deviations. Regarding pairwise comparisons (Table 2), visual, olfactory, and overall rating significantly differed between the INFO-SENS group compared to the SENS group, while the remaining comparisons were not statistically significant.

To further investigate whether these effects depend on prior NoLo knowledge, a composite Liking score was calculated as mean of visual, olfactory, gustatory, and overall assessment. This score was submitted into a two-way ANOVA examining the main effects of information (INF), prior knowledge (KNO), and their interaction (INF × KNO). The results satisfied the homogeneity of variances assumption according to Levene’s test and showed significant effects of INF (F = 9.645, *p* = 0.002), KNO (F = 12.979, *p* < 0.001), and INF × KNO (F = 10.634, *p* = 0.001) on Liking. Post hoc comparisons indicated a compensatory pattern effect in the interaction between prior knowledge and experimental information (Table 3). Prior experience in the absence of information (INF− KNO+) was associated with lower Liking compared with the INF− KNO−condition. By contrast, information alone without prior knowledge (INF+ KNO−) did not differ significantly from INF− KNO−. However, information improved Liking among the experienced participants: INF+ KNO+ outperformed INF− KNO+, effectively counterbalancing the negative effect of prior expectations. Likewise, INF+ KNO− also outperformed INF− KNO+.

Finally, to explore whether consumption habits and socio-demographic characteristics were associated with Liking, we estimated a linear regression model with Liking as the dependent variable and gender, age group, citizenship, wine consumption, wine color preferences, prior NoLo knowledge and tasting experience as predictors. Given the exploratory nature of these analyses and the modest sample size for some subgroups, the results are interpreted cautiously. The results reported in Table 4 indicate that in our experimental conditions Liking was generally not associated with consumption habits, including regular wine consumption, preferences for specific wine types (red, white, rosé), and prior NoLo tasting experience. In contrast, socio-demographic variables such as gender and non-EU citizenship showed significant effects on Liking, whereas age does not exhibit a statistically significant association.

### 3.3. Willingness-to-Pay Across Experimental Conditions

Given the markedly non-normal distribution of WTP [Shapiro–Wilk (W = 0.840, *p* < 0.001)] and significant heteroscedasticity [Levene’s (F = 10.635, *p* < 0.001)], a Kruskal–Wallis test was employed as the primary one-way analysis, with Welch-corrected ANOVA applied as a complementary robustness check. Both tests confirmed significant differences in WTP across the experimental groups. Mean WTP was highest in the INFO group (M = 5.52, SD = 1.64), followed by the INFO-SENS group (M = 4.71, SD = 2.52) and the SENS group (M = 3.81, SD = 2.75). The larger standard deviation observed in the SENS condition suggests that, in the absence of an informational anchor, the participants expressed more heterogeneous price expectations. Post hoc comparisons (Table 2) based on Dunn’s test showed that WTP was significantly lower in the SENS group than in the INFO group (Δ = −1.698, *p* < 0.001) and significantly higher in the INFO-SENS than in the SENS group (Δ = 0.895, *p* = 0.033). The INFO-SENS and INFO groups did not differ significantly under Dunn’s test (Δ = −0.803, *p* = 0.164), whereas the corresponding Welch-corrected comparison reached only marginal significance (*p* = 0.097). In summary, the statistics reveal a relatively weak contrast between INFO-SENS and INFO, but a clearer distinction can be observed between the informed and uninformed groups, consistent with a model in which information acts as a reference frame for economic evaluation.

To further investigate the relationship between sensory appreciation and economic valuation, a Spearman rank correlation was computed between Liking and WTP, confirming a significant positive association (ρ = 0.525, *p* < 0.001, N = 187). Nevertheless, Liking was generally associated with greater economic valuation but did not exclusively determine it. This suggests the presence of other independent drivers of WTP, further explored through the Tobit regression model. The use of this model is methodologically appropriate given the left-censored distribution of the outcome variable, as it accounts for the structural mass at zero arising from the participants who expressed no purchase intention (n = 32; 17% of the sample) [48,49].

The Tobit regression model was estimated to examine the joint influence of information exposure, prior NoLo wine knowledge, and individual-level characteristics on WTP (Table 5). Two models were estimated: Model 1 tested the main effects, while Model 2 included the interaction between information exposure and prior knowledge (INF × KNO), consistent with the two-way ANOVA approach.

**Table 5:** Tobit coefficients (β) and Average Marginal Effects (AME) for willingness-to-pay across experimental conditions, socio-demographic characteristics, consumption habits and NoLo experience.

	Model 1		Model 2	
Levels of Variable	β (*p*-Value)	AME (*p*-Value)	β (*p*-Value)	AME (*p*-Value)
Liking	1.064 (0.000)	1.043 (0.000)	1.015 (0.000)	0.996 (0.000)
Male	0.067 (0.849)	0.066 (0.848)	0.047 (0.892)	0.046 (0.892)
Italian	0.196 (0.730)	0.192 (0.730)	0.313 (0.577)	0.308 (0.577)
Not-UE	0.341 (0.771)	0.334 (0.771)	−0.257 (0.824)	−0.252 (0.824)
Age < 45	0.008 (0.982)	0.007 (0.982)	−0.170 (0.625)	−0.167 (0.625)
Wine_consumer_yes	0.463 (0.407)	0.454 (0.406)	0.538 (0.329)	0.528 (0.327)
Red	−0.821 (0.050)	0.804 (0.049)	−0.831 (0.045)	−0.815 (0.043)
White	−0.127 (0.730)	0.125 (0.729)	−0.173 (0.635)	−0.170 (0.634)
Rosè	−0.331 (0.406)	0.325 (0.405)	−0.154 (0.698)	−0.152 (0.698)
Information (INF+)	1.234 (0.001)	1.210 (0.001)	-	-
Knowledge (KNO+)	−0.719 (0.090)	−0.705 (0.089)	-	-
INF− KNO−	-	-	2.393 (0.002)	2.348 (0.002)
INF+ KNO−	-	-	3.056 (0.000)	2.998 (0.000)
INF+ KNO+	-	-	3.024 (0.000)	2.968 (0.000)
Prior NoLo Tasting_yes	−0.212 (0.731)	−0.020 (0.730)	−0.361 (0.553)	−0.355 (0.553)

Note. Model 1 includes the main effects of information exposure (INF) and prior NoLo knowledge (KNO) and is reported as a robustness specification. Model 2 adds the interaction between INF and KNO using INF− KNO+, as baseline. Other reference categories used as baseline: female, EU citizenship, age ≥ 45. WTP was estimated with a Tobit model to account for censoring at 0; N uncensored = 155, limits: 0–9.

Both Tobit models were globally significant and showed satisfactory fit indices. In Model 1, Liking (β = 1.064, *p* < 0.001) and information exposure (β = 1.234, *p* = 0.001) were the only significant positive predictors of WTP, whereas prior knowledge of NoLo wines showed a negative, marginal effect (β = −0.719, *p* = 0.090). Model 2 provided a more detailed test of the interaction INF × KNO, which yielded results consistent with the compensatory pattern observed for Liking. Using INF−KNO+ as the reference category, prior NoLo knowledge was associated with lower WTP in the absence of information, while information exposure attenuated this negative effect. The highest WTP was observed for the INF+KNO− group (β = 3.056, *p* < 0.001), followed closely by INF+KNO+ (β = 3.024, *p* < 0.001) and INF−KNO− (β = 2.393, *p* = 0.002).

Across both models, red wine preference had a significant negative effect (β = −0.831, *p* = 0.045), suggesting that stronger attachment to conventional red wine may reduce the economic valuation of its dealcoholized counterpart. All remaining individual-level covariates—including gender, age, citizenship, wine consumption frequency, and previous tasting experience—did not reach conventional significance thresholds (all *p* > 0.10). The negative intercept indicated that a hypothetical consumer with all covariates set to zero would have a latent WTP below the purchase threshold, consistent with the proportion of non-buyers in the sample. Conditional marginal effects computed at variable means showed that each additional unit of Liking was associated with an increase of €1.04 in expected WTP among buyers, while information exposure remained the second significant positive determinant (AME = €1.21), indicating that the informed participants were willing to pay more than uninformed peers independently of their sensory evaluation.

## 4. Discussion

### 4.1. Information as a Driver of Sensory Evaluation and Economic Valuation (H1)

The results obtained by one-way ANOVA strongly support H1, indicating that product information contributes not only to economic valuation but also, albeit more moderately, to sensory appreciation. However, this pattern should be interpreted with some caution, as the groups were broadly comparable to most demographic characteristics but differed in wine consumption status. Information on both production characteristics and nutritional aspects appears to have improved sensory evaluations of dealcoholized wine in a selective way. In particular, visual, olfactory, and overall assessment were higher in the INFO-SENS group compared to the SENS group, whereas gustatory ratings did not differ significantly across conditions, possibly also reflecting specific sensory characteristics of the single experimental wine that limited between-group differences in palate-related evaluations.

This pattern suggests that some sensory dimensions—particularly those related to visual and olfactory appraisal—may be more responsive to expectation-based framing, while palate-related perceptions remain less susceptible to cognitive modulation and may be more closely linked to alcohol-related mouthfeel and structure, often perceived as thinner and less complex [13].

However, the product was not rejected on sensory grounds, as overall assessment remained moderately positive across the groups, ranging from 6.23 to 6.98, indicating sensory acceptability despite dealcoholization. This likely reflects production practices adopted for the wine sample, such as low-temperature vacuum evaporation and the replacement of pasteurization with sterile microfiltration, which help limit organoleptic losses.

Although previous studies suggest that alcohol content remains one of the main determinants of perceived quality and price in NoLo beverages [35], skepticism toward the product persists in Italy [42], and alcohol reduction may trigger a symbolic devaluation even when sensory appreciation remains acceptable [24,50]. In this context, clear and credible communication regarding production processes, grape origin, and preserved wine characteristics becomes essential to reduce resistance in sensory perception, particularly with respect to visual and olfactory appraisal, as well as production technology.

Information also appears to act as an anchoring effect, playing a role in shaping perceived economic value, with the INFO group reporting the highest WTP and the lowest standard deviation compared to the SENS group. The absence of a further increase in the INFO-SENS condition suggests that information may have already created a cognitive frame. Once expectations were established through information, sensory tasting seems to have provided only limited incremental value in influencing economic judgment.

Moreover, the absence of informational cues (SENS) seems to amplify the negative impact of sensory evaluation on the economic assessment of dealcoholized wine. This pattern aligns with the theory of expectation disconfirmation, whereby extrinsic cues shape expectations, perceived quality and purchasing behavior before direct sensory confirmation occurs [27,28,29], in contrast to Masson & Aurier [51] who observed that NoLo wines are less likely considered “real wine” as alcohol content decreases, independently of both sensory and non-sensory evaluation conditions. Our results suggest that information may help in reframing dealcoholized wine as a legitimate innovation rather than an inferior traditional wine, consistent with Angelini et al. [32], who reported that cumulative information increases consumers’ willingness-to-pay in wine evaluation contexts.

### 4.2. Prior Knowledge and the Role of Information (H2)

Given the pivotal role of information in shaping both willingness-to-pay and sensory evaluation (H1), we further examined whether prior knowledge of NoLo wine moderates its effect on sensory appreciation and WTP. The results are particularly noteworthy and partially support H2, indicating that prior NoLo knowledge alone does not necessarily enhance acceptance; rather, it appears to activate more critical evaluation schemas. However, when prior NoLo knowledge is combined with information (INF × KNO), this negative effect is effectively reversed among knowledgeable participants, suggesting a compensatory effect rather than a simple substitution effect.

This finding suggests that knowledgeable consumers may activate more critical schemas linking dealcoholization to quality loss, which can translate into lower sensory scores and WTP. In contrast, novice consumers display greater openness to novelty, with higher levels of acceptance and minimal need for cognitive reframing. This interaction extends H1 by showing that information operates interactively with pre-existing cognitive schemas. In this sense, prior knowledge may generate skepticism barriers rather than fostering acceptance based on familiarity [52], whereas information can operate as a corrective interpretative framework that reshapes perceptions of the product, particularly within Italian wine culture, which remains highly sensitive to issue of authenticity [25,26].

These findings are broadly consistent with the literature showing that NoLo consumers may perceive the category as lower tier when they are more familiar with its limitations. At the same time, they contrast with evidence suggesting that the evaluation of novel products is driven primarily by intrinsic sensory characteristics [53], or that non-sensory cues such as low-alcohol labeling alone do not necessarily generate significant differences in consumption behavior among regular red wine drinkers when comparing NoLo and standard wine [54].

Communication about product details also appears to interact with sensory evaluation. Informed consumers tend to provide more positive ratings, whereas uninformed consumers may lack the cognitive framework needed to interpret the product more favorably. For consumers already familiar with NoLo wines, communication strategies may be more effective when emphasizing preserved aromatic quality, varietal identity, and the retention of bioactive compounds. This mechanism also mirrors patterns observed in price formation, where premium quality attributes tend to generate the strongest price premiums in upper market segments, suggesting that more knowledgeable consumers are better able to recognize value in quality-oriented categories [55].

### 4.3. Wine Habits and Socio-Demographic Characteristics Influence Sensory Evaluation and Willingness-to-Pay for Dealcoholized Wine (H3)

A particularly relevant finding is that, unlike previous studies indicating that younger consumers or women tend to be more open to dealcoholized wine [23,41], our controlled design reduces most demographic differences, thereby highlighting information exposure and prior knowledge as the dominant drivers of both sensory evaluation and economic valuation. This interpretation is further supported by evidence showing that cognitive and attitudinal variables are often more informative than broad socio-demographic segmentation in explaining consumer heterogeneity [56].

The linear regression model predicting Liking showed that wine consumption habits did not exert any significant effect on sensory appreciation, whereas socio-demographic characteristics such as gender and non-EU citizenship reached significance, providing only partial support for H3. These findings, considered jointly with those supporting H1 and H2, suggest that familiarity-related factors, information exposure, and prior knowledge are the primary drivers of sensory appreciation for dealcoholized wine, while economic valuation is more strongly shaped by experimental conditions than by individual characteristics.

H3 is further partially supported by the Tobit regression results, which confirm the dominance of informational cues in predicting WTP. Socio-demographic variables were either non-significant or showed only weak and non-robust effects, with the exception of red wine preference, which exhibited a borderline negative association with WTP.

Within this framework, Liking emerged as the strongest predictor of WTP, showing a strong and positive association with economic valuation, as expected. However, the marginal effect of information on WTP, estimated ceteris paribus and therefore net of sensory appreciation, directly addresses the core research question: information outweighs sensory evaluation in shaping the economic value attributed to dealcoholized wine.

While sensory evaluation remains relevant for assessing product quality, it does not translate into proportionally higher economic value when information is simultaneously available. The comparison across experimental conditions clearly indicates that information is the primary driver of WTP, regardless of whether tasting occurs before or after valuation, thereby refining the interpretation of H1.

Although prior analyses suggested that information may indirectly enhance WTP through improved sensory perception, particularly via visual and olfactory dimensions, the results indicate that the effect of information is not fully explained by Liking. Rather, information appears to operate through a distinct cognitive pathway, influencing perceived value, quality expectations, and product legitimacy independently of taste-based responses. From a broader perspective, this helps explain a central tension emerging from the study: even in the presence of favorable sensory evaluations, information triggers a process of economic re-evaluation rather than simply reinforcing hedonic preferences. This mechanism is especially salient for dealcoholized wine, a product embedded in a culturally sensitive category strongly associated with authenticity, alcohol content, and traditional production norms. In such contexts, consumer responses are shaped not only by intrinsic sensory attributes but also by the symbolic and cognitive meanings activated through informational cues [57].

## 5. Conclusions

This study examined how product information and consumer sensory evaluation shape acceptance of partially dealcoholized red wine in an Italian sample context, focusing on WTP, sensory appraisal, prior NoLo knowledge, and consumer characteristics. Overall, product information emerged as the main driver of economic valuation and, to a lesser extent, of sensory appreciation. Information and prior knowledge acted jointly in shaping consumer responses, suggesting that cognitive framing can partially compensate for skepticism toward dealcoholized wine. In practical terms, transparent communication about production methods, grape composition, origin, processing technology and potential health benefits may help consumers to interpret the product more positively, thereby enhancing acceptance and payment behavior [58]. At the same time, the study suggests that positive sensory tasting does not necessarily translate into an equal increase in economic evaluation. This gap indicates that, for this category, acceptance depends not only on taste but also on symbolic and informational cues linked to authenticity and product legitimacy. Producers should therefore combine improvements in sensory quality with clear and credible communication, especially when addressing consumers with different levels of familiarity with NoLo products.

In contrast to other non-alcoholic beverages, such as beer, where taste remains the primary determinant of repurchase intention [59], for wine such information may act as perceptual priming, shaping expectations and directing amplifying attention to sensory cues [60]. Communication strategies should also be differentiated across consumer segments: for knowledgeable consumers, emphasis on aromatic quality, varietal identity, and retained bioactive compounds may be more effective, whereas for less experienced consumers, novelty and lifestyle-oriented framing may better support adoption [57,61].

Despite these opportunities, the future development of dealcoholized wine in Italy will also depend on the evolving regulatory and institutional framework, which may represent both a constraint and an opportunity for producers [62]. In this perspective, broader market diffusion will require not only technological improvement and consumer education, but also coherent policy support and transparent labeling standards with sustainability-oriented approaches and collaborative networks, in order to build an infrastructure of trust and innovation that individual producers cannot sustain on their own [63].

This study has some limitations. The experimental sensory setting does not fully reproduce real consumption contexts, where bottle presentation, brand cues, social environment, and purchase occasion may influence evaluation. The analysis focused on a single red dealcoholized wine, obtained experimentally and characterized by a specific compositional profile that may have influenced sensory appreciation, thereby limiting the generalizability of the findings across other wine types. In addition, WTP was elicited using a payment card method which, although widely used, may be affected by hypothetical bias. Accordingly, WTP should be interpreted as a stated, hypothetical valuation rather than observed market behavior. A further limitation is that the information sheet presented production, nutritional, and process-related cues simultaneously; therefore, the present design does not allow disentangling the relative effect of each informational component on sensory appraisal or WTP.

Future research should extend the analysis to different wine categories and more realistic consumption settings, including field and retail-based experiments. Further investigation into repeated exposure effects, alternative information framing, and underlying psychological mechanisms would also help clarify how cultural context shapes consumer acceptance. Overall, this study contributes to the emerging literature on NoLo wines by demonstrating that acceptance in Italy depends not only on sensory quality but also on the informational and symbolic conditions under which the product is experienced, highlighting a pathway for market development based on the integration of technological improvement, effective communication, and institutional support.

## Figures and Tables

**Table 1:** The descriptive statistics for socio-demographic characteristics and wine-related variables across the three experimental groups.

Variables	Levels	Total (n = 187)	INFO-SENS (n = 59)	SENS (n = 60)	INFO (n = 68)
Gender	Male	94 (50.3%)	33 (55.9%)	28 (46.7%)	33 (48.5%)
	Female	93 (49.7%)	26 (44.1%)	32 (53.3%)	35 (51.5%)
Age Range	18–29	69 (36.9%)	17 (28.8%)	27 (45.0%)	25 (36.8%)
	30–44	49 (26.2%)	19 (32.2%)	12 (20.0%)	18 (26.5%)
	45–59	50 (26.7%)	19 (32.2%)	13 (21.7%)	18 (26.5%)
	≥60	19 (10.2%)	4 (6.8%)	8 (13.3%)	7 (10.3%)
Citizenship	Italian	164 (87.7%)	50 (84.7%)	56 (93.3%)	58 (85.3%)
	EU	18 (9.6%)	5 (8.5%)	4 (6.7%)	9 (13.2%)
	Non-EU *	5 (2.7%)	4 (6.8%)	0 (0.0%)	1 (1.5%)
Wine Consumer	Yes *	152 (81.3%)	51 (86.4%)	42 (70.0%)	59 (86.8%)
	No	35 (18.7%)	8 (13.6%)	18 (30.0%)	9 (13.2%)
Wine Preference	Red	100 (53.5%)	33 (55.9%)	32 (53.3%)	35 (51.5%)
	White	61 (32.6%)	22 (37.3%)	17 (28.3%)	22 (32.3%)
	Rosé	65 (34.8%)	20 (33.9%)	20 (30.0%)	25 (36.8%)
Prior NoLo Knowledge	Yes	52 (27.8%)	14 (23.7%)	16 (26.7%)	22 (32.4%)
	No	135 (72.2%)	45 (76.3%)	44 (73.3%)	46 (67.6%)
Prior NoLo Tasting	Yes	20 (10.7%)	10 (16.9%)	4 (6.7%)	6 (8.8%)
	No	167 (89.3%)	49 (83.1%)	56 (93.3%)	62 (91.2%)

Note. Values are frequencies and column percentages. Superscripts refer to chi-square tests for between-group comparability: * variables with significant differences across groups (*p* < 0.10).

**Table 2:** Mean (SD), one-way ANOVA and post hoc pairwise comparison for sensory rating and willingness-to-pay (EUR per 125 mL) across experimental groups.

Variables	Descriptives			Statistics
	INFO-SENS (n = 59)	SENS (n = 60)	INFO (n = 68)	
Visual	7.831 (1.224) ^A^	7.183 (1.760) ^B^	7.441 (1.123) ^AB^	Welch F = 3.629 **
Olfactory	6.712 (1.921) ^A^	5.783 (1.941) ^B^	6.294 (1.861) ^AB^	F *=* 3.545 **
Gustatory	6.237 (2.104) ^A^	5.850 (2.223) ^A^	5.765 (1.886) ^A^	F = 0.911
Overall Rating	6.983 (1.581) ^A^	6.233 (1.969) ^B^	6.426 (1.586) ^AB^	F *=* 3.070 **
WTP	4.71 (2.519) ^A^	3.817 (2.752) ^B^	5.515 (1.644) ^A^	H *=* 13.104 **

Note. Descriptives are means and standard deviations (SD) for each experimental group. For sensory attributes (visual, olfactory, gustatory, overall rating), differences across the groups were tested using one-way ANOVA; when Levene’s test indicated heteroscedasticity, Welch’s corrected F was used (as indicated in the Statistic column). Different capital letters within the same row indicate significant pairwise differences at *p* < 0.10 based on post hoc comparisons (Tukey HSD or Games–Howell, depending on variance homogeneity). Means sharing at least one letter do not differ significantly. For WTP, the primary one-way test was the Kruskal–Wallis H-test because of non-normality and censoring at zero; post hoc pairwise comparisons were performed with Dunn’s test, and letters reflect these non-parametric post hoc results. Significance levels: *p* < 0.05 (**).

**Table 3:** Post hoc pairwise comparisons of the interaction INF × KNO on Liking.

Group		Mean Difference	SE	t	df	p_tukey_ ^1^
INF− KNO−	INF+ KNO−	−0.039	0.260	0.149	183	0.999
	INF− KNO+	1.709	0.413	4.139	183	<0.001 (***)
	INF+ KNO+	0.124	0.318	0.389	183	0.980
INF+ KNO−	INF− KNO+	1.670	0.383	4.357	183	<0.001 (***)
	INF+ KNO+	0.085	0.278	0.306	183	0.990
INF− KNO+	INF+ KNO+	−1.585	0.425	−3.721	183	0.001 (***)

^1^ Tukey HSD post hoc. Significance levels: *p* < 0.01 (***).

**Table 4:** OLS regression coefficient (β) for Liking by socio-demographic characteristics, wine consumption habits and NoLo experience.

Variables	Levels	β	Std. Error	*p*-Value
Gender	Female	0.564 **	0.215	0.009
Age Range	Age < 45	0.052	0.216	0.811
Citizenship	Italian	−0.003	0.354	0.994
	Non-EU	1.380 *	0.735	0.062
Wine Consumer	Yes	−0.296	0.348	0.396
Wine Preference	Red	−0.179	0.259	0.491
	White	0.351	0.229	0.126
	Rosé	−0.477 *	0.246	0.054
Prior NoLo Knowledge	Yes	−0.656 **	0.257	0.012
Prior NoLo Tasting	Yes	0.002	0.380	0.995

Note. Dependent variable: Liking (composite score of sensory variables). Coefficients from OLS regression, Standard Error and *p*-values. Reference categories: male, age ≥ 45, EU citizenship, non-consumers, no NoLo wine knowledge, no prior NoLo tasting experience. Significance levels: *p* < 0.1 (*), *p* < 0.05 (**).

## Data Availability

The original contributions presented in this study are included in the article. Further inquiries can be directed to the corresponding author.
